# Assessment of the Microbiological Quality of Groundwater in Three Regions of the Valencian Community (Spain)

**DOI:** 10.3390/ijerph110505527

**Published:** 2014-05-22

**Authors:** Agustín Llopis-González, Adriana L. Sánchez, Pedro Martí Requena, María Morales Suárez-Varela

**Affiliations:** 1 Unit of Public Health, Hygiene and Environmental Health, Department of Preventive Medicine and Public Health, Food Science, Toxicology and Legal Medicine, University of Valencia, 46010 Valencia, Spain; E-Mails: agustin.llopis@uv.es (A.L.-G.); asanver2@alumni.uv.es (A.L.S.); 2 CIBER Epidemiologia y Salud Pública (CIBERESP), 28029 Madrid, Spain; 3 Mix Unit of Nutrition and Public Health, Centre for Public Health Research (CSISP), 46010 Valencia, Spain; 4 Public Health Laboratory, Centre for Public Health Research (CSISP), 46010 Valencia, Spain; E-Mail: martirequena@yahoo.es

**Keywords:** groundwater, *E. coli*, groundwater bodies, microbiological contamination, vulnerability maps

## Abstract

Urban groundwater development was traditionally constrained by concerns about its quality. This study was conducted in the regions of La Ribera Alta and Ribera Baja and La Plana de Requena-Utiel of the Valencian Community (Valencia, Spain) where population density, demand for drinking water and agricultural activities are high. Groundwater bodies (GWBs) are regarded as management areas within each territory, and were used to establish protection policies. This study analyzed eleven GWBs. We used two databases with microbiological measurements from 154 wells over a 7-year period (2004–2011), risk factors and groundwater information. Wells were grouped according to frequency of microbiological contamination using *E. coli* measurements, category <1, or wells with low-frequency microbiological contamination and high-frequency wells or category 1–100, according to World Health Organization (WHO) quality criteria of drinking water. Of all wells, 18.12% showed high-frequency microbiological contamination with a majority distribution in the Ribera Alta region (26.98%, *p* < 0.001). No significant differences were found between the two risk categories for flow, static level, well depth and distance from population centres. This paper reveals that the vulnerability classes established by the Geological and Mining Institute of Spain (IGME) do not match the microbiological results, and that only eight wells with high-frequency contamination coincide with the high vulnerability areas.

## 1. Introduction

Water is vital for all living organisms to survive, and for the functioning of ecosystems, communities and economies worldwide. It is essential and indispensable for all human activity and for all forms of life. Preserving quality is important for drinking water supply, food production, and for maintaining living systems, ecological and recreational use, among others.

Water quality worldwide is increasingly threatened, which spells a threat for the health of people and ecosystems, reduces availability of drinking water and water for other uses, thus productivity and development opportunities will suffer. Among the risk factors affecting water quality, we find a wide variety of human and natural processes: agriculture, industry and energy production, mining, uncontrolled human waste disposal, population growth, urbanisation and climate change, which all affect surface and groundwater resources.

In Spain, many populations use groundwater supplies, and it is necessary to assess their quality to establish appropriate protection methodologies in the most vulnerable areas for both by the aquifers intrinsic characteristics and external risk factors associated with human activities.

The Valencian Community is one of the Spanish regions where water is a limited resource, which has become one of the main problems for both its socio-economic development and the environmental quality of its lands today and tomorrow; hence the need to ensure water quality and to take measures to ensure the conservation of groundwater resources.

The European Union established the COST Action 620 for vulnerability mapping and risk of contamination to protect carbonated aquifers (karst), which could be adapted into appropriate methods to be used in individual karst areas of Europe [1]. Vulnerability maps are the result, and they show areas in different colours, depending on the degree of vulnerability, so they are easy to interpret and can be used as a practical tool for land use planning and protection zones. In Spain, the Geological and Mining Institute Spain (IGME) used the COP method [2] for carbonated aquifers and the DRASTIC method [3] for detrital and mixed aquifers. Both methods are based on geological, hydrological and hydrogeological characteristics.

The purpose of this study was to assess the quality of eleven (11) groundwater bodies (GWB) in the Júcar River Basin (CHJ) area (Valencia, Spain) according to microbiological measurements. This study also focuses on the relationship between risk factors and contamination of wells. Finally we compared the results obtained with the classes of vulnerability to contamination determined by the IGME according to the methodology proposed by the European Union through COST Action 620 for protecting groundwater against pollution and deterioration.

## 2. Experimental Section

### 2.1. Test Site Characteristics

The study area is located in the regions of Ribera Alta, Ribera Baja and Plana de Requena-Utiel of Valencian Community (Valencia, Spain). It covers a surface area of 3,018 km^2^, with a population of 339,658 [4]. The Plana de Requena-Utiel region is located in the west province of Valencia between the central plateau and the coast. Dominated by vineyards, which are the basis of regional agriculture, it is an agricultural region and one of the largest wine-producing areas. The Ribera Alta consists of a broad valley, crossed by the Júcar River. From its slopes, a vast plain extends to the sea and is interrupted only by the presence of the Ribera Baja and the Sierra de la Murta and Agulles near the coast. The Ribera Baja region is characterized by a flat area, flanked on its right by the Sierra de Corbera, with altitudes of over 500 m. In this region, rice crops extend into the southern part of the Albufera Lagoon, with orange crops.

### 2.2. Groundwater Bodies (GWB)

**Figure 1:**
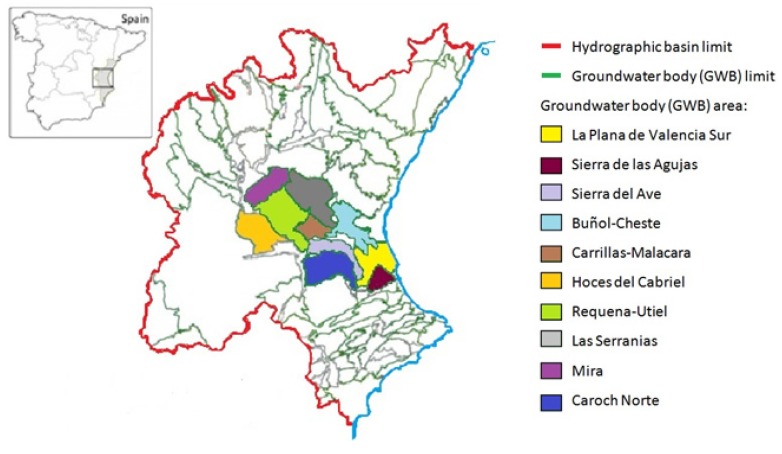
The groundwater bodies considered in the study.

The work unit normally used to establish management criteria is the Exploitation System, which corresponds to uniform areas formed by surface water and GWBs. GWBs are aquifers or aquifer sets, which can be considered management areas within each territory. The largest territory in Valencia belongs to the Júcar River Basin (CHJ), except in the northwest, which corresponds to the Ebro basin, and to the south end, which corresponds to the Segura Basin [5]. The GWBs covered by this study are: La plana de Valencia Sur, Sierra de las Agujas, Sierra del Ave, Buñol-Cheste, Carillas-Malacara, Hoces del Cabriel, Requena-Utiel, Las Serranías, Mira and Caroch Norte (Figure 1).

### 2.3. Database

We used two databases provided by the Centre for Research on Public Health (CSISP), which took microbiological data measurements between 2004 and 2011 of 154 wells distributed in three regions in Valencia. During this period, several measurements were taken in a single well per year, and one measurement per month was taken, but monthly averaging was done.

The databases containing information on location of wells, microbiological analysis of total coliforms, *E. coli*, aerobic at 22 °C, foecal streptococci (FE), sulphite reducing clostridium (SRC) and geographical coordinates of the .well, risk factors associated with each one, proximity to population centres, flow, depth and static level. The presence/absence of the following factors in wells was considered: cased, fencing and shed, within a radius of 90 m around the well were considered solid waste landfills, cesspools, sewers, water waste, farms and irrigated land.

Microbiological measurements were taken by standard membrane filtration techniques and the results were confirmed by presence/absence in samples. Groundwater information was collected using the Júcar River Basin map viewer. Given lack of information, such as geographic coordinates, 16 wells were eliminated from the study and 138 wells were analysed.

### 2.4.Data Grouping

Wells were grouped according to frequency of microbiological *E. coli* contamination. Grouping into risk categories was done based on the World Health Organization (WHO) drinking water criteria, which takes into account *E. coli* cultures to be an indicator of pollution [6]. In the category <1, that is, low-frequency contaminated wells, those wells that exceeded the WHO Drinking Water criteria less than 25% of the times sampled were grouped [7]; high-frequency contaminated wells were considered those in category 1–100, and exceeded the WHO quality criteria by 25% or more of the times sampled.

### 2.5. Statistical Analysis

The microbiological data, the risk factors (fenced, shed, solid waste landfill, cesspool, sewer, wastewater, farms and irrigation) and geographical data were stored in an Excel database. Data were exported for further analysis using the Epi Info [8] and SPSS v.13 (SPSS Inc., Chicago, IL, USA) statistical packages. The initial analysis used ordinal data. The continuous variables with normal distribution were expressed as mean ± SD and categorical variables were expressed as percentages. Differences in the various regions (Utiel, Requena, Ribera Alta and Ribera Baja) were evaluated by a Chi-Square test for the categorical variables and an analysis of variance (ANOVA) for the continuous variables. Data were analysed using SPSS package procedures (SPSS v.19; IBM Corporation, Chicago, IL, USA). All the tests were 2-tailed and a *p* value threshold of <0.05 was set for statistical significance.

### 2.6. Vulnerability Maps

The flow concentration, overlying layers and precipitation (COP) [9] and Depth to groundwater, net recharge, aquifer media, soil media, general topography or slope, vadose zone, hydraulic conductivity of the aquifer (DRASTIC) [3] methods are based on the Pan-European Approach to intrinsic vulnerability mapping proposed by COST Action 620. The COP method was applied in Spain for carbonated aquifers and the DRASTIC method was used for detrital and mixed aquifers.

The intrinsic vulnerability index COP method takes into account three factors indicated by COST Action 620 as factor C (flow Concentration) and the surface conditions controlling the water flowing towards rapid infiltration zones, which are less capable of attenuating contamination; factor O (overlying layers), which provides protective groundwater layers; and factor P (precipitation), on precipitation characteristics. The COP vulnerability index is obtained by multiplying the three factors and from the resulting index, five vulnerability classes are obtained, ranging between 0 and 15, where values close to 0 indicate maximum vulnerability (minimum protection), and those close to 15 correspond to high vulnerability (maximum protection) [10].

For the DRASTIC vulnerability index and mixed detrital aquifers, the IGME proposed using only four (4) of the seven (7) variables used in the original method given the difficulty arising from the limited information available from the unsaturated zone [10]. These variables are: Soil media (S), upper portion of the unsaturated zone with biological activity; Lithology of the unsaturated zone (L) reflects the flow conditions that influence the time available for adsorption processes, reactivity and dispersion lead to changes in contaminant; thickness of the unsaturated zone (E), soil layer will have to cross before reaching the contaminant piezometric surface of unconfined aquifers; net recharge (R) water capacity can carry contaminants into the aquifer. In the application of DRASTIC methods, many studies have dropped or added factors based on contaminant characteristics, study purposes and landscape backgrounds. For instance, Li and Merchant (2013) [11] dropped Impact of vadose zone (I) and aquifer hydraulic conductivity (C), and added a land use factor for the groundwater vulnerability modelling. Huan *et al.* [12] used similar approach to modify the DRASTIC model for their groundwater vulnerability study as well. The result of this method is expressed on a scale from one (1) to ten (10), where 1 is the lowest score and 10 represents maximum vulnerability. To interpret the results, we grouped the scale values. Values of 1 and 2 mean very low susceptibility values, 3 and 4 mean low values, 5, 6 and 7 are intermediate values, while 8, 9, 10 are high vulnerability.

## 3. Results

According to the risk categories based on the *E. coli* measurements, 81.88% (*p* < 0.001) of wells meet the WHO guidelines for drinking water (<1 UFC/100 mL) and 18.12% wells show high-frequency contamination (1–100 CFU/mL). Wells are distributed mainly in the La Ribera Alta region, where 26.98% (*p* < 0.001) correspond to wells with a high risk of contamination (Table 1).

Microbiological quality measurements were taken for 258 wells in category <1. The average value was 0.12 for *E. coli* (Table 2). Category 1–100, the total number of measurements was 60 and the average value was 4.02 *E. coli* CFU/mL. It should be noted that standard deviations were very high for the two risk categories, and that no statistical significance was found between the means of the two categories (*p* < 0.001). The analyses considered foecal streptococci (FE) and sulphite reducing clostridium (SRC) in each well, detected in at least one (1) measurement. FE were detected in 64.00% of the wells with a high risk of contamination, while these pathogens were detected were detected in only 13 wells (11.50%) of the 113 wells with a low risk. SRC was also observed in a higher percentage (36.00%) of wells positive for high risk *vs.* 6.19% of wells positive for low risk (Table 2).

**Table 1:** Distribution of wells according to location and risk categories.

Categories	Criteria	No. Wells	Percentage (%)	Region						
				Utiel Requena		Ribera Alta		Ribera Baja		*p*
				No. Wells	%	No. Wells	%	No. Wells	%	
<1	Low frequency	113	81.88	47	92.16	46	73.02	20	83.33	0.030
1–100	High frequency	25	18.12	4	7.84	17	26.98	4	16.67	0.030
All wells		138	100	51	36.96	63	45.65	24	17.39	<0.001
*p*			<0.001		<0.001		<0.001		<0.001	

**Table 2:** Pathogenic bacteria and *E. coli* according to the risk categories.

Categories	No. samples	No. Wells	*E. coli*				Percentage of positive wells	
			Mean	SD	Min	Max	FE	SRC
1	258	113	0.12	1.99	0.00	32.00	11.5	6.19
1–100	60	25	4.02	10.00	0.00	55.00	64	36
All wells	318	138	--	--	--	--	21.01	11.59
*p*	--	--	--	--	--	--	<0.001	<0.001

The average depth of the wells with a high risk of contamination was 126.0 m, and their water level is shallower (39.8 m) than the levels of the wells with less risk (48.8 m). Wells with a high risk have a higher flow rate (3,104.9 L/min) than those at low risk (2,271.5 L/min). Regarding proximity to population centres, the wells with a low risk were located further away from a population centre (Table 3), but these parameters in the wells showed no statistical significance between the two groups of wells.

**Table 3:** Profile characteristics wells.

Criteria (Well)	Categories								Statistical analysis of difference
	<1				1–100				
	Mean	SD	Min	Max	Mean	SD	Min	Max	
Depth (m)	140.83	108.37	0.00	560.00	126.04	87.40	4.00	300.00	t = 0.62; *p* = 0.472
Static level (m)	48.79	63.66	0.00	400.00	39.75	33.69	0.00	120.00	t = −0.95; *p* = 0.343
Flow (L/min)	2,272.29	2,490.08	0.00	14,166.00	3,102.08	3,590.89	0.00	15,000.00	t = 1.10; *p* = 0.281
Proximity to population centr (m)	1,572.29	2,064.99	0.00	10,000.00	1,300.0	1,750.70	0.00	5,000.00	t = −0.69; *p* = 0.492

**Figure 2:**
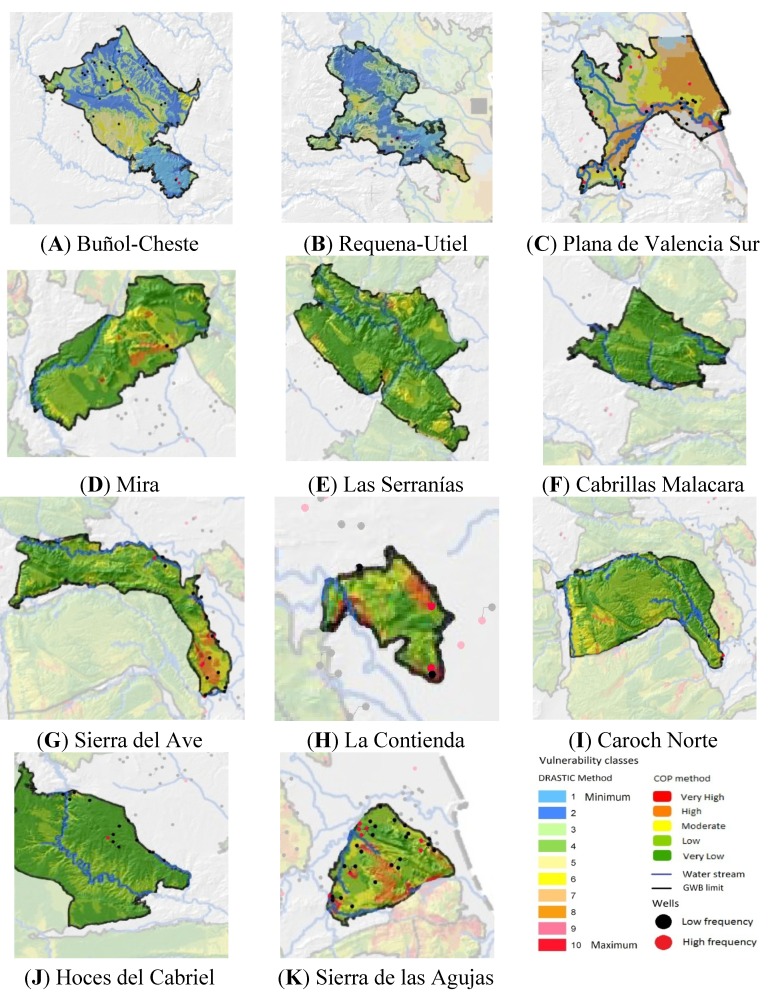
Distribution of wells by groundwater bodies (GBW) (**A**) Buñol-Cheste; (**B**) Requena-Utiel; (**C**) Plana de Valencia Sur; (**D**) Mira; (**E**) Las Serranías; (**F**) Cabrillas Malacara; (**G**) Sierra del Ave; (**H**) La Contienda; (**I**) Caroch Norte; (**J**) Hoces del Cabriel; (**K**) Sierra de las Agujas.

The distribution of GWBs and vulnerability classes was determined by the IGME (Madrid, Spain) classification to protect the groundwater used for drinking water according to the DRASTIC and COP methods (Table 4). Of the 113 wells grouped in category <1 (low risk of contamination), 51 wells were located in carbonated GWBs and 62 wells in detrital and mixed GWB. A statistically significant difference was observed between the percentage of wells in the low and high risk of contamination categories for the carbonated GWBs (*p* < 0.001) and for the detrital and mixed GWBs (*p* < 0.001). Of the 25 wells with a high risk of microbiological contamination, 14 (56%) were found in carbonated GWB and 11 in detrital or mixed GWBs (Figure 2). Of all the wells in carbonated GWBs, 42.85% (35.71% and 7.14%) are catalogued with vulnerability among low and very low and 57.13% (8 wells) were located in areas of high and very high vulnerability. Among the detrital and mixed GWBs, there was no well with high vulnerability, and most wells corresponded to intermediate categories (Table 4).

**Table 4:** Distribution of wells for groundwater body (GWB) type and category.

Categories	GWB type and vulnerability class										
	Carbonated *						Detrital-Mixed *				
	No. wells	Very high	High	Moderate	Low	Very Low	No. wells	High (8,9,10)	Intermediate (5,6,7)	Low (3,4)	VeryLow (1,2)
<1	51	9.8	25.49	29.41	9.8	25.49	62	9.67	40.32	40.32	9.67
1–100	14	21.42	35.71	0	35.71	7.14	11	0	63.63	9.09	27.27
All wells	65	--	--	--	--	--	73	--	--	--	--

* Expressed as percentage of group.

When analysing the different risk factors, having a protective shed in the well was not significant, but a significant difference was found between the percentage of wells not fenced (64.00%, *p* = 0.048) and the percentage of those with such protection. A high percentage (84.00%, *p* < 0.001) of wells were located within a 90-m distance from cesspools. Farms in the vicinity of the wells, solid waste landfills or wastewater sources other than septic tanks or sewers were not found (Table 5). According to the statistical analysis, presence of sewers in the area where a well is located did not prove significant for risk of contamination, which is a contradictory result to that found for presence of cesspool. A high percentage of wells (76%, *p* < 0.001) were no cased, which may facilitate the entry of pathogenic bacteria through a direct interaction between soil and water. This situation, along with proximity of cesspools proximity and lack of fenced wells, increase vulnerability and can be considered important factors that affect the microbiological quality of water.

**Table 5:** Risk factors for wells with high frequency of microbiological contamination.

Risk factors of Contamination *	Risk factors (expressed as percentage)								
	Cased (type of well)	Fenced	Shed	Distance < 90 m					
				Solid waste landfill	Cesspool	Sewer	Wastewater	Farms	Irrigation
Yes	24.00	36	40		84	56			24
No	76.00	64	52	88	4	32	88	100	76
*p*	<0.001	0.048	0.392		<0.001	0.087			<0.001

* Presence of farm, cesspools, solid waste landfills and population.

## 4. Discussion

### 4.1. Microbiological Contamination

This study provides evidence for microbiological contamination in carbonated and detrital aquifers in the region of Valencia. According to the *E. coli* measurements in 138 wells between 2004 and 2011, 18.12% of the wells present high frequency microbiological contamination according to the WHO quality criteria for drinking water (Table 1). These results are consistent with those reported in other studies, where aquifers with similar characteristics have been analysed. In aquifers of Uganda [13], high concentrations of foecal pathogens to be associated with domestic wastewater and presence of sedimentary rocks have been reported. Other authors [14] have linked the use of manure in agriculture and soil type with high levels of coliform in groundwater in limestone/dolostone rock soils in areas of Canada and Zimbabwe. In the UK, first time evidence for the depth and extent of microbiological contamination of groundwater deriving from sewage pollution in two types of sandstone aquifers in Birmingham and Nottingham has been presented [15]; this study was carried out over a 15-month period and showed that distinct types of foecal bacteria and viruses can penetrate different depths in association with seasonal variations and presence of preferential flow paths, such as fissures. In Yemen, *E. coli* concentrations of up to 106 CFU/mL have been found in alluvial aquifers [16].

Pathogenic bacteria, like foecal streptococci (FE) and sulphite reducing clostridium (SRC), were detected in both groups of wells, especially for the high-frequency contamination wells. Detection of SRC in nine (9) wells of 25 (36.00%) indicates that these bacteria can survive in unsaturated zones of aquifers, which were introduced into unsaturated zones through heavy rainfall or irrigation to reach groundwater, as previous studies have indicated [17]. In 64% of the high-frequency contamination wells, FE were identified, which has been regarded to be a more reliable indicator that presence of *E. coli*.

It has been reported that the estimated total and focal coliforms are not sufficient to confirm foecal contamination. Therefore, the estimation of FE and SRC was included. It is known that FE are more resistant than coliforms in natural environments, and that they can be an indicator of foecal contamination [18] and of resistance due to clostridial spores, which allows them to survive for long periods of time. The analyses of these two pathogens (EF and SRC) confirmed the contamination state of the wells showing a high frequency of microbiological contamination. Moreover, their presence in low-risk wells may be due to water source discharges or residues with high microorganism loads of foecal origin.

### 4.2.Spatial Distribution of Wells

We found that most of the wells in the high-risk category (68.00%) were located in the Ribera Alta region (Table 1). This region has the highest population, 218,482 [4], and is distributed in 35 municipalities whose population centres exert stronger pressure on ater requirements for human consumption and to undertake certain activities such as agriculture, which predominates in this area, and this implies a greater volume of wastewater.

### 4.3.Risk Factors

Different aspects relating to the characteristics of well construction, location and hydrology were compared for both well groups. Among the two risk categories, the analysis indicated that for depth, flow, static level and proximity to population centres, there was no statistically significance between the two groups of wells (Table 3). However, these results may have been affected by lack of records for a considerable number of wells. Yet despite this, and based on different studies [6], we should not rule out the possibility that distance to population centres can be considered an important risk factor since, in this study, 10 of the 25 high-risk wells are located in densely populated areas or on their periphery.

The depths of the wells at high risk of contamination ranged from 0 to 300 m, with an average depth of 126 m if compared to 140.8 m for the low-risk category wells (Table 3). This may indicate that deeper wells have an increased ability to filter contaminants through different soil layers. It should be noted that some studies have reported pathogenic bacteria at a depth of 36 m [19] and others [15] at 91 m in confined aquifers.

Apart from proximity to urban areas, 76.00% of the wells (Table 5) were not cased (*p* < 0.001), which increases the possibility of pathogenic microorganisms contaminating wells by different transport mechanisms, as formerly described by several authors [15,20,21]. This transport is facilitated by the presence of fractures, pore size, and discontinuities are preferred routes for microorganisms in vertical and horizontal directions. In the non-cased wells, no restricted water intake and microorganisms can enter at any depth, while for cased wells, microorganisms tend to fall deeper and an increased interaction exists with soil and microorganisms being absorbed by soil particles, with a consequent reduction in pollutant load [14].

When considering the influence of protection of wells, no statistical significance was found for wells with a shed. However, a significant difference was observed between the percentage of wells with no fencing (64.00%, *p* = 0.048) and the proportion with such protection (36%). Therefore, this protection can be considered an important risk factor.

There was a high percentage (84.00%, *p* < 0.001) of wells at high risk of contamination located at a distance of less than 90 m from a cesspool (Table 5). This result coincides with several studies, which have evaluated the influence of untreated wastewater on groundwater discharge into the ground. In Algeria, seven areas where wastewater was discharged without treatment have been evaluated, to find well water samples with foecal coliform up to 210 CFU/mL [22]. Other authors have concluded that the main source of pollution in Kampala (Uganda) is local surface discharge and wastewater [13], as well as by the presence of latrines, which are sites of frequent foecal waste disposal in developing countries. A study conducted in Nebraska (USA) has determined that the wells located within 30 m of a septic system with water depth below 14 m show very high vulnerability to microbiological and chemical contamination [23].

### 4.4. Type of Groundwater Body (GWB)

Of the wells with a high risk of contamination, 14 were located on carbonated GWBs and 11 on detrital and mixed GWBs (Table 4). These results do not match the affirmation that carbonated aquifers have higher vulnerability to other aquifer types [10]. Most studies done in the European Union [2,24,25] have focused on the evaluating the quality of carbonated aquifers because protection has been prioritised through European actions such as COST 620.

This study provides evidence for vulnerability to different microbiological contamination of GWB than those considered by the IGME and the COST Action 620 as priorities for protection activities [1]. Some wells present evident microbiological contamination in detrital and mixed GWBs (Table 4), suggesting that vulnerability to contamination should not focus exclusively on carbonated aquifers or be generalized to the GWB type with these characteristics because all aquifers have some degree of vulnerability to pollution, and they depend not only on soil properties, but also on other factors such as those deriving from human activities.

### 4.5.Maps and Classes of Vulnerability to Pollution

This study shows that vulnerability classes do not coincide with the microbiological results (Table 4) and of the 25 wells at high risk of contamination, only eight coincide with high vulnerability area. Hence we can state that there is no direct relationship between the vulnerability category and frequency of microbiological contamination for the wells considered in this study.

These results suggest the need to evaluate the information provided by maps of vulnerability to contamination determined by the COP and DRASTIC methods to provide verified information that is useful for planning and decision making for groundwater protection purposes. Different methodologies have been proposed to evaluate this mapping which use different measurements types in the field; among them we find the measurement of microbiological parameters, and the tracer technique that has been used in Sierra de Líbar [24] in an aquifer in Spain into which three dyes (eosin, uranine, sulphorhodamine B) were injected through three holes in the ground and sampled wells in the area, This information was used to validate the information of the intrinsic vulnerability map, and the most vulnerable areas coincided with the areas that were detected by tracers.

Intrinsic vulnerability maps have the disadvantage of considering only the ability of soil to protect groundwater from contamination and do not consider dangerous activities resulting from human activity. For this reason, it is necessary to use specific methodologies that consider both the geological and hydrological characteristics of the area as particular properties of contaminants. Thus, it will be possible to establish safeguard zones for the supply wells located on any type of GWB and in areas where direct communication occurs with aquifer area.

According to the literature search, no studies have been found in which the microbiological quality of aquifers relate with the vulnerability classes established by the COP and DRASTIC methods. This evaluation is important because the future activities proposed to prevent water quality deterioration are based on this classification and they focus on the areas classified as high and very high vulnerability which, according to the results of this study, risk of contamination does not necessarily coincide with these areas, which would leave unprotected areas with a significant risk of contamination.

## 5. Conclusions

This study shows that the groundwater in the Utiel-Requena, Ribera Alta and Ribera Baja Regions of the Valencian Community (Valencia, Spain) are vulnerable to microbiological contamination due to risk factors such as human activities, lack of well protection structures and the hydrogeological characteristics in the area. In public health terms, recognising this vulnerability is clearly important in areas where groundwater is the main source of drinking water. The microbiological analyses do not fully relate with the specific vulnerability classes as a result of implementing COP and DRASTIC for the protection of groundwater used for drinking water according to the Water Framework Directive requirements.
